# Normalization of boutique two-color microarrays with a high proportion of differentially expressed probes

**DOI:** 10.1186/gb-2007-8-1-r2

**Published:** 2007-01-04

**Authors:** Alicia Oshlack, Dianne Emslie, Lynn M Corcoran, Gordon K Smyth

**Affiliations:** 1 Walter and Eliza Hall Institute of Medical Research, Royal Parade, Parkville, Victoria, Australia

## Abstract

Normalization is critical for removing systematic variation from microarray data. For two-color microarray platforms, intensity-dependent lowess normalization is commonly used to correct relative gene expression values for biases. Here we outline a normalization method for use when the assumptions of lowess normalization fail. Specifically, this can occur when specialized boutique arrays are constructed that contain a subset of genes selected to test particular biological functions.

## Abstract 2

A new normalization method is described for use in specialized boutique arrays which contain a subset of genes selected to test particular biological functions.

## Background

Normalization of microarray data is the process of removing systematic bias and variation caused by technical artifacts while maintaining the important biological variation of interest. After appropriate normalization, variation in microarray data should be unbiased with respect to the samples being compared. As normalization is performed to adjust relative intensities between samples, microarray studies are most effective when looking at expression differences between samples rather than expression differences between genes. The extent of normalization required for an experiment depends on the quality and consistency of the arrays and samples being compared. Different microarray platforms require different strategies but the most widely used methods are intensity dependent. It has been shown that alternative normalization procedures can have substantial effects on results for a variety of platforms [1-3]. For two-color microarrays, intensity-dependent lowess normalization has emerged as a general purpose method and is the most commonly used procedure for normalization.

Lowess normalization attempts to correct the expression log-ratios for inequalities between the labeling dyes. The relative preponderance of one dye over the other often changes with the intensity of the measurements. Therefore the fit to the expression log-ratios of the two cannels (M) is performed against the average log-intensity of the two cannels (A) i.e. on an MA-plot [4]. Effective lowess normalization relies on the assumption that either: the majority of genes are not differentially expressed; or there is symmetry in the expression levels of the up and down regulated genes [5]. Furthermore, as the procedure is intensity dependent it requires a sufficient number of genes with these properties at the full range of intensities. These assumptions are typically very reasonable for large-scale genome arrays because differences between RNA samples will typically relate to molecular pathways involving only a small proportion of the entire genome. The assumptions can fail, however, in a range of special scenarios related to the biology of the samples being compared or the probes being tested. In such a situation, it is not clear how best to normalize arrays.

This article considers the case of focused custom arrays that are printed with a relatively small number of selected probes of particular interest. These boutique arrays, featuring from a few score to several hundred genes, can have advantages over genome-wide arrays for expression profiling. Although there are far fewer genes overall, the coverage is often increased for the specific gene family or pathway of interest. Moreover, because there are fewer irrelevant probes, the specificity of the arrays is increased, resulting in a lower false discovery rate. Boutique arrays can, therefore, be used as a moderately high throughput assay to systematically interrogate genes of maximal interest at low cost [6,7]. Boutique arrays are almost always two-color cDNA arrays because cDNA arrays are the easiest to customize and the least expensive to print in-house.

Boutique arrays do pose special problems for normalization. The lowess curve may be unreliably estimated because there are relatively few distinct probes from which to estimate the curve. Furthermore, as the genes on the arrays are preselected to be of interest, there is no reason to expect the genes to be evenly distributed over the intensity range or to be unbiased with respect to the expression levels in the samples. It is quite possible that more than half the probes might be differentially expressed between any two samples and that the differential expression might be predominately in one direction. Therefore, the assumptions required for standard lowess normalization commonly fail.

There is as yet no widely accepted standard method for normalization of boutique arrays. Studies using custom arrays have utilized a variety of methods, including standard lowess normalization [7], normalization by house keeping genes [8-10], total intensity or global normalization [2,5,11] and normalization using spike-in controls [12]. This article shows that all of these methods can produce biased results. Dye-swap normalization has also been suggested as a method for normalizing arrays with a high proportion of differentially expressed genes [13], but such methods require multiple arrays to perform any normalization and are not adapted to small boutique arrays. The use of normalization genes with balanced differential expression has recently been proposed for normalizing small diagnostic arrays [14]. Although this method addresses the issue of normalizing microarrays with a small number of biased probes, it is limited to comparing a pair of RNA sources that are known in advance. It is not available for differential expression arrays designed to compare a variety of RNA sources

A titration series of a whole microarray transcript pool (MSP) has been proposed as a way to construct unbiased control probes for normalization purposes [5]. In this article, we observe that the transcript pool need not be constructed from the probes on the array to be normalized but may instead be constructed from a much larger transcript library for the same species. This article demonstrates the effectiveness of transcript pool control probes for normalizing boutique arrays. A new method for utilizing such control probes that introduces probe-specific quantitative weights into the lowess normalization procedure is proposed. As far as the authors are aware, this is the first use of quantitative weights in intensity-dependent normalization. The weighted lowess method is shown to provide a flexible, reliable and accurate normalization method for boutique microarrays.

## Results and discussion

### Robustness of lowess normalization

We begin by investigating the robustness of lowess normalization, a topic which was mentioned in the original lowess publication but which has not been explored in the literature. Robustness refers to the ability of a statistical technique to follow the major trend of a dataset and to ignore outlier values. Robustness of lowess normalization means that it can tolerate some asymmetry in differential expression between the samples being hybridized provided that the majority of genes are not differentially expressed [5]. What is not clear is how large the proportion of differentially expressed genes can be before lowess becomes unsuitable for normalization. A small simulation study is sufficient to verify the robustness property and to demarcate its limitations.

Data were taken from a self-self hybridization of a two-color microarray containing 11,088 probes. These data were not expected to show differential expression as the same sample was hybridized to both channels. The data were background corrected and lowess normalized and then used for our simulations.

**Figure 1:**
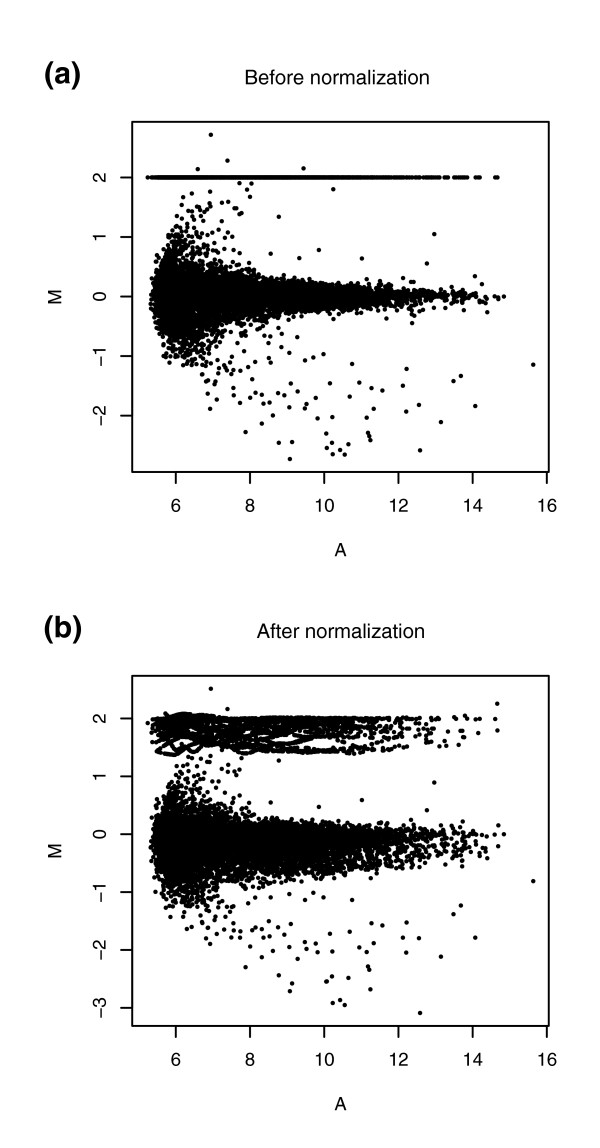
Breakdown of lowess normalization. An MA-plot for a self-self hybridization is shown with a set of randomly selected genes designated to be differentially expressed at *M *= 2 or four-fold up-regulated. Results for 25% of genes on the array randomly being up-regulated are shown **(a) **before and **(b) **after print-tip lowess normalization. After normalization the fold change for most up-regulated genes has been reduced and bias has been introduced into the non-differentially expressed genes.

We simulated an extreme case where the lowess assumptions are most likely to fail. Genes were randomly assigned to have large differential expression values in only one direction. The designated genes were set to have log_2 _ratios of two, that is, to be four-fold up-regulated (Figure 1a). The data were then renormalized using print-tip lowess normalization [5] (Figure 1b) and these artificially up-regulated genes were assessed for their stability of up-regulation. The proportion of artificially unregulated genes was varied and the normalization assessed for robustness.

**Figure 2:**
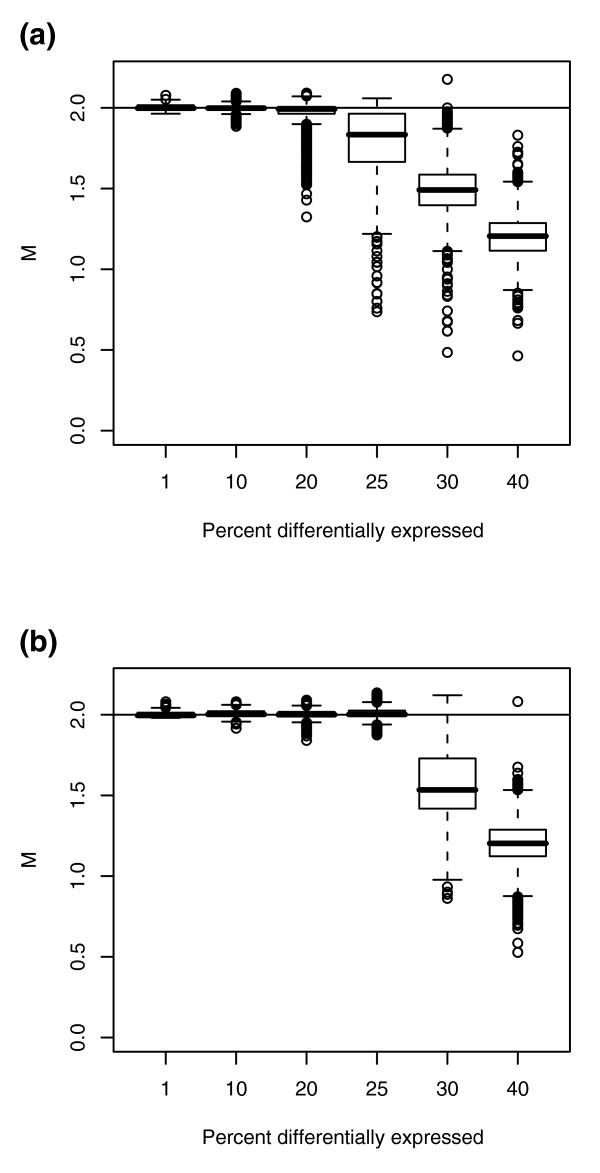
Boxplots of up-regulated genes after normalization. Each box plot represents the *M *values of the up-regulated genes after print-tip normalization as the percent of genes differentially expressed is varied. If the normalization procedure is robust to the outliers then *M *= 2. **(a) **In this scenario print-tip lowess is robust to approximately 20% of differentially expressed genes when the default number of iterations is equal to 3. **(b) **For 10 robustifying iterations the normalization is reliable for approximately 25% of genes differentially expressed.

The results are shown in Figure 2, where the box plots represent the log_2 _differential expression of the up-regulated genes as the percentage of up-regulated genes is varied. If lowess normalization is performing well, then these box plots should be consistently at a log differential expression of two. In this example it can be seen that the stability of lowess normalization holds even when approximately 20% of genes are 4-fold up-regulated (Figure 2a). There is a robust iteration step in lowess normalization (see Materials and methods). If the number of robustifying iterations in the lowess fit is increased from the default of three iterations to ten, the algorithm becomes more robust to outliers. Figure 2b shows that this increases the tolerance of lowess normalization from approximately 20% to approximately 25% of up-regulated genes. We repeated this procedure using global lowess normalization rather than print-tip dependent normalization and found that the mean results were similar, although the variance was decreased (data not shown).

Given these results, lowess normalization will be appropriate for many applications even if up to 20% of genes show asymmetric differential expression. This shows that the domain of applicability of lowess normalization is wider than is sometimes characterized [13]. When the percentage exceeds this figure, other normalization techniques will, in general, be required, such as the one presented in the next section. The figure of 20% could be extended if the differential expression is not all in one direction, but the degree of symmetry of differential expression is uncertain in boutique arrays, which are our focus here.

### Normalization of boutique arrays

Boutique arrays are custom-made arrays that may contain only a few score genes. With such small arrays it is easy to step beyond the tolerance of lowess normalization, particularly as genes are often selected on the basis of having a changing role in the samples being compared. A natural way to normalize these arrays is to train the lowess curve on a set of control probes that should not change between samples. Several types of controls have been suggested for this purpose, including housekeeping genes, spike-in controls and microarray sample pool (MSP) controls.

#### Housekeeping genes

Housekeeping genes are thought to have expression levels that are biologically so tightly regulated that they will not change between samples. The appeal of these for normalization purposes is, therefore, obvious and has been widely used. However, true housekeeping genes are hard to come by and many that have previously been used for normalization have been shown to be differentially expressed between samples or treatments [15,16].

#### Spike-in controls

**Figure 3:**
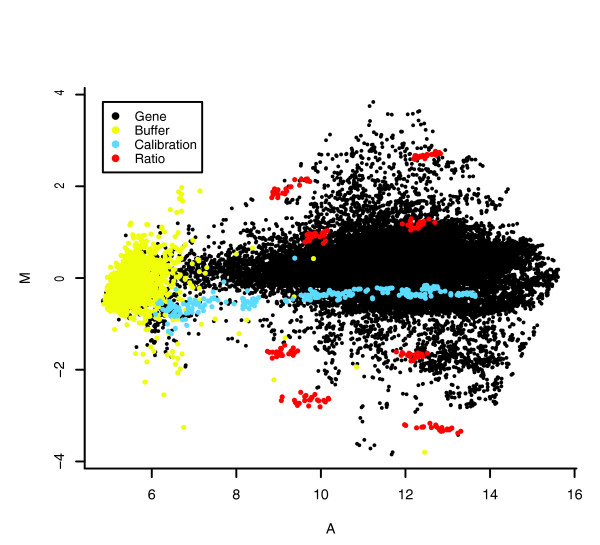
Normalization curve using spike-in controls. MA-plot with spike-in controls indicated. The blue points represent calibration controls where equal amounts of foreign RNA have been spiked into each sample. Red points represent ratio controls at three-fold and ten-fold differential expression. It can be seen that the spike-in probes are significantly offset from the gene probes and, therefore, could not be used for normalization purposes.

Spike-in controls involve printing a set of foreign controls onto the arrays and then adding their corresponding transcripts into the RNA before labeling and hybridization. If genes are spiked in at the same concentration then they should not be differentially expressed and, therefore, should be useful for normalization [12]. The fact that the spike-in RNA is not extracted with the main RNA sample and has to be added separately means that the spike-in probes will not always follow the same intensity-dependent normalization curve as the regular probes. This is illustrated in Figure 3, which shows an array where the spike-in controls are clearly offset from the locus of gene probes and, therefore, would need to be normalized independently.

#### Microarray sample pool control

cDNA microarrays are typically printed from a library of cloned cDNA samples. An MSP is constructed from a clone library by combining all the members of the library together in equal quantities to make a heterogeneous pool. The pool is then diluted to give a range of five to ten different concentrations. These titrated MSP samples are then spotted onto the slides several times, giving probes with a range of intensities similar to the intensity range in genes of interest. The library from which the pool is constructed must be sufficiently large for the assumption of no average differential expression between samples to be reasonable. The MSP has the effect of simulating the average expression that would be observed on a microarray constructed from the entire library, and so will have the essential properties required for lowess normalization. This construction has previously been shown to be not differentially expressed between closely related samples [5]. We suspect that a pool containing as few as 500 randomly selected genes will have the desired characteristics for many applications. This figure is derived from extensive experience with print-tip lowess normalization on arrays for which the print-tip groups contain around 400 spots. Unlike spike-in control spots, MSP probes do not require foreign RNA to be added to the samples. MSP controls interact instead with the RNA from the samples themselves, which are the quantities to be normalized.

#### Composite normalization using MSP probes

**Figure 4:**
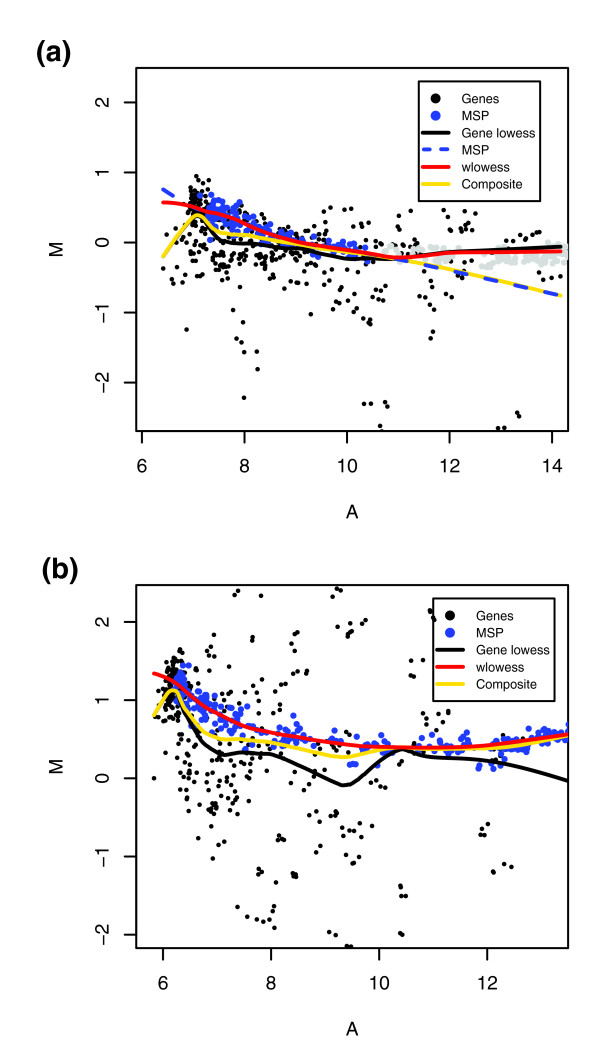
Normalization using composite normalization. MA-plots with a lowess normalization curve for the gene probes only (black), for the composite normalization (yellow) and for the MSP weighted lowess (red) with the MSP probes shown in blue. **(a) **The MSP probes do not extend to the full intensity range and the composite normalization curve follows the extension of the MSP only curve. **(b) **It can be seen that at low intensities the majority of gene probes are down-regulated compared to MSP probes, meaning that the lowess curve and, therefore, the composite normalization curve are biased downwards compared to the MSP.

Yang *et al*. [5] outlined a composite normalization strategy in which adjustment was a weighted average of the lowess fit to the MSP probes and a lowess fit to the gene probes. The gene probe fits were estimated for each print-tip group. The proportion of the contribution from the lowess fits for each probe type changed with intensity such that more weight was given to the fit of the gene probes at low intensities while the fit to the MSP probes had more weight at high intensities. This method was used successfully on comparisons of medial versus lateral portions of the olfactory bulb [17]. This method is not generally appropriate for boutique arrays as it requires a sufficient number of unbiased probes at low intensities where the lowess curve generated from the gene probes has the largest influence on the normalization adjustment. Problems at extremities of intensities can also occur if gene probes reach higher or lower intensities than the MSP probes. Figure 4 shows an example of a boutique array where the normalization curve is generated using the composite normalization strategy. As this array contains a very small number of gene probes, we did not use spatial (print-tip) normalization to construct the gene probe lowess but instead used all gene probes in a global lowess. Figure 4a demonstrates how composite normalization behaves if the full range of intensities for the MSP probes is not available. It can be seen that, at high intensity, composite normalization follows the trend given by the MSP probes, which is continued regardless of the curvature of the data. Figure 4b shows that the composite method is biased towards the gene probes at low intensities. In this example the gene probes are, on average, down-regulated compared to the MSP probes.

#### Weighted lowess normalization

We propose the use of MSP probes to normalize custom arrays in an alternative way to the composite normalization strategy that will be robust against probe selection bias at all intensities. In this strategy information from all the probes is used to perform lowess normalization but MSP probes are given more weight across the entire intensity range compared to gene probes. We call this weighted lowess normalization procedure 'wlowess'. The up-weighted lowess curve is also shown in Figure 4b, where the wlowess curve is comparatively unbiased at low intensities. The up-weighting of the MSP probes in relation to the gene probes can be quite significant such that they dominate the fitted curve. However, the use of information from all probes provides a solution for when the MSP probes do not cover the full range of intensities. In these situations the wlowess curve follows the curve generated by the gene probes (Figure 4b), which at least represents the curvature of the data.

This new approach extends the lowess smoothing procedure by defining a set of quantitative weights that are applied to each of the probes. The estimation of this curve on an MA-plot is then used for normalization. The use of quantitative weights allows control probes to be up-weighted relative to gene probes. Moreover, it ensures that the normalization will smoothly make optimal use of whatever mixture of control probes and gene probes are available on an intensity-dependent basis. For very small boutique arrays it is unlikely that there will be enough probes in each print-tip group to perform print-tip lowess normalization. Nevertheless, the procedure can be easily extended to print-tip normalization providing that a range of MSP probes are printed with each print-tip. The Materials and methods section gives a description of the lowess procedure and the extension to wlowess.

### B-lymphocyte boutique arrays

**Figure 5:**
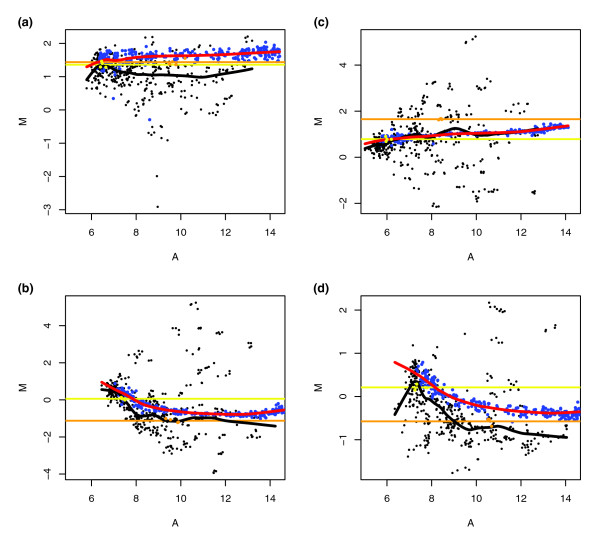
Normalization comparison for a boutique array. MA-plots for four examples of a boutique array designed to profile 109 genes during the late stages of B-lymphocyte differentiation. Each cDNA clone is spotted on the array four times (black points). The MSP titration series are shown as blue points. The black line is the lowess fit through the gene probes only. The red line is the weighted lowess fit with MSP probes up-weighted as described in the methods. The yellow and orange points and lines correspond to the differential expression levels of two house keeping genes, *HPRT *and *GAPDH*, respectively. **(a, b, d) **show examples where the gene probe lowess and the wlowess curves are considerably different from each other. **(b, c, d) **show examples where house keeping genes give very different intensities from each other and from the wlowess curve.

We illustrate the wlowess method using a boutique array designed specifically to profile differentially expressed genes during the late stages of B-lymphocyte differentiation. On these arrays 109 genes of interest have each been spotted four times. Comparisons at different stages of differentiation with different growth factors have been made as part of a larger experiment. In Figure 5 we show four examples of these arrays that illustrate the normalization technique. The MSP probes are shown in blue and the red line is our weighted lowess fit to the data. If we ignore the MSP probes and perform a lowess fit to the gene probes, the black curve is generated. For Figures 5a, c, d there are substantial differences between the two lowess curves, which can be separated up to two-fold in differential expression at some intensities. This is caused by an asymmetric distribution of differential expression for the majority of genes on the arrays, causing ordinary lowess normalization to fail.

Figure 5 also indicates the differential expression of two putative housekeeping genes, those encoding glyceraldehyde 3-phosphate dehydrogenase (GAPDH) and hypoxanthine phosphoribosyltransferase 1 (HPRT). The four replicate probes are shown with a straight line through the mean of the probes as estimates were only made at one intensity level for each gene. These two genes can diverge in expression up to two-fold (Figure 5b-d). Even when MSP weighted lowess and regular lowess agree, house keeping genes can still diverge (Figure 5c).

Often it is easy to assess the general accuracy of a normalization procedure by looking at MA-plots before and after normalization [4,5]. By nature, boutique arrays contain a small number of probes that are possibly biased, making it difficult to assess whether a normalization method has been successful. The introduction of a set of unbiased probes such as a MSP can be used for unbiased normalization. We suggest that information for all probes be used in the lowess fit by making use of probe-specific numerical weighting.

## Conclusion

We have demonstrated through a series of examples that previous methods of normalization of boutique microarrays can commonly bias results. We introduce a weighted lowess normalization method using spot specific quantitative weights to up-weight MSP probes compared to gene probes. This produces unbiased differential expression for the whole range of intensities. The 'wlowess' method can be used on any two-color arrays where the probe selection is thought to be biased. It can be used not only with MSP controls but with any subset of probes that are known *a priori *not to be differentially expressed between samples. The weighted lowess method is extremely flexible, being capable of adapting to a range of situations in which alternative methods may fail. Even when the situation is suitable for related methods, such as ordinary lowess or composite lowess, the weighted lowess method is never worse. The lowess weights can also be used to down-weight lower quality spots or to remove known differentially expressed probes, such as spike-in ratio controls, which should not be included in the normalization. The functions to carry out this normalization procedure are implemented in the bioconductor [18] package limma [19]. The MSP controls can be constructed from any large scale transcript library for the species under consideration, so the MSP material can be prepared in bulk in a cost-effective manner for wide-spread use in a range of experiments. In principle, appropriate MSP material could be provided commercially in the same way that spike-in kits are currently offered.

## Materials and methods

### Lowess normalization

Robust locally weighted regression referred to as lowess or loess is a nonparametric procedure widely used for smoothing scatter plots [20]. For normalization of two-color arrays it is used for robust smoothing of MA-plots. For each spot *i *on an array two measurements are made, the intensity of the Cy5 or red channel (*R*_*i*_) and the intensity of the Cy3 or green channel (*G*_*i*_). An MA-plot is a difference-mean plot where the *M *values are the log ratio of red to green for each probe:

*M*_*i *_= log_2 _*R*_*i *_- log_2 _*G*_*i*_

and the *A *values are the average log intensity of the two channels [4]:

*A*_*i *_= 0.5(log_2 _*R*_*i *_+ log_2 _*G*_*i*_)

The normalized values are *M*_*i *_- *f*(*A*_*i*_) where *f*(*A*_*i*_) is the lowess curve through the points.

The normalization method developed here also uses the lowess procedure developed by [20] to estimate *f*(*A*_*i*_) but introduces spot specific quantitative weights. In general, the lowess smoothing function is generated as follows.

A first order polynomial is fitted to the *M*_*j *_on the *A*_*j *_with distance weights:

*d*_*ij *_= *d*(|*A*_*i *_- *A*_*j*_|)

using weighted least squares. Here *d*() is a decreasing function that is exactly zero for all *j *outside a neighborhood of *A*_*i*_. The neighborhood is defined as a proportion of points with *A*_*j *_closest to *A*_*i *_called the span. The span is typically set to 0.3 to 0.4 [3]. The value of the polynomial at *j *= *i *becomes *f*(*A*_*i*_).

Subsequent iterations use robust weights:

*r*_*i *_= *r*(|*M*_*i *_- *f*(*A*_*i*_)|)

where *r*() is another decreasing function giving large weight to small residuals and small weight to large residuals for each point. The polynomial is refit by weighted least squares to obtain *f*(*A*_*i*_) but now with weights *d*_*ij*_*r*_*j*_. Three robustifying iterations are typically used and the final values for *f*(*A*_*i*_) are subtracted from the *M*_*i *_for normalization.

We introduce prior weights *w*_*i *_associated with each probe or spot. In each stage of the polynomial fitting procedure the weighted least squares estimation incorporates these weights. In the first step the weights become *w*_*j*_*d*_*ij *_and in the robustifying steps the fitting is done using the weights *w*_*j*_*d*_*ij*_*r*_*j *_to estimate *f*(*A*_*i*_) for all *i*. The values of *w*_*i *_can be defined by the user. Typically, for MSP normalization purposes we define *w*_*i *_= 1 for MSP probes and *w*_*i *_= 0.01 otherwise. This methodology can also be used to down-weight suspect or low quality spots on individual arrays.

### Boutique B-cell microarrays

A boutique microarray collection, comprising 109 probes, was created and spotted on to glass slides. The probes were PCR fragments corresponding to cDNAs for genes known to be differentially expressed during late stages of B lymphocyte differentiation either from the literature or from our own investigations using semi-quantitative PCR. Three housekeeping genes were also included. Each probe in the collection was printed four times on the arrays. MSP probes were created by pooling the clones of the NIA15K cDNA library [21]. The MSP was prepared at different dilutions to make concentrations of 250 ng/μl, 120 ng/μl, 60 ng/μl, 30 ng/μl, 15 ng/μl, 7 ng/μl, 4 ng/μl, 2 ng/μl and 1 ng/μl. Each concentration was printed 32 times on the arrays to make a total of 288 MSP control spots. Differential hybridizations were performed using cDNAs synthesized from sorted populations of activated B lymphocytes, from *in vitro *derived plasmablasts, or from terminally differentiated plasma cells sorted directly *ex vivo*. The cells were from OBF-1^-/- ^knock-out mice [22] and C57BL/6 control mice. Pairwise (competitive) hybridizations included analogous populations from controls versus mutants, or developmentally related populations within a strain (for example, undifferentiated versus differentiated).

Raw data used in this paper can be found at [23].
